# Excessive femoral torsion is not associated with patellofemoral pain or instability if TKA is functionally aligned and the patella denervated

**DOI:** 10.1007/s00167-022-07162-5

**Published:** 2022-09-16

**Authors:** Andreas Flury, Armando Hoch, Gabriele Cirigliano, Sandro Hodel, Nathalie Kühne, Stefan M. Zimmermann, Lazaros Vlachopoulos, Sandro F. Fucentese

**Affiliations:** Orthopaedic Department, Balgrist University Hospital, University of Zurich, Forchstrasse 340, CH-8008 Zurich, Switzerland

**Keywords:** Femoral torsion, Patellofemoral pain, Total knee arthroplasty

## Abstract

**Purpose:**

Recent data suggest that individual morphologic factors should be respected to restore preoperative patellofemoral alignment and thus reduce the likelihood of anterior knee pain. The goal of this study was to investigate the effect of excessive femoral torsion (FT) on clinical outcome of TKA.

**Methods:**

Patients who underwent TKA and complete preoperative radiographic evaluation including a long-leg radiograph and CT scan were included. 51 patients showed increased FT of > 20° and were matched for age/sex to 51 controls (FT < 20°). Thirteen patients were lost to follow-up. Thirty-eight matched pairs were compared after a 2 year follow-up clinically (Kujala and patellofemoral score for TKA) and radiographically (FT, frontal leg axis, TT-TG, patellar thickness, patellar tilt, and lateral displacement of patella). Functional alignment of TKA was performed (hybrid-technique). All patellae were denervated but no patella was resurfaced.

**Results:**

There was no significant difference between clinical scores two years after surgery between patients with normal and excessive FT (n.s.). Kujala score was 64.3 ± 16.7 versus 64.8 ± 14.4 (n.s.), and patellofemoral score for TKA was 74.3 ± 21 versus 78.5 ± 20.7 (n.s.) for increased FT group and control group, respectively. There was no correlation between preoperative FT and clinical scores. Other radiographic parameters were similar between both groups. No correlations between clinical outcomes and preoperative/postoperative frontal leg axis or total leg axis correction were found (n.s.).

**Conclusion:**

If the leg axis deformity is corrected to a roughly neutral alignment during cemented TKA, including patellar denervation, then excessive FT was not associated with patellofemoral pain or instability.

**Level of evidence.:**

Prospective comparative study, level II.

## Introduction

With up to 10%, anterior knee pain is the most common reason for unsatisfying outcomes following total knee arthroplasty (TKA), and leads the ranking of reasons for TKA revision surgery [2, 34, 43]. According to literature, including national registry data, 25–30% of all TKA revisions are performed because of patellar problems [27, 28, 40].

Patellofemoral maltracking can occur in both resurfaced and non-resurfaced patellae [29]. An important technical consideration to avoid patellofemoral maltracking includes appropriate rotational component alignment [30]. In addition, recent data suggest that individual morphologic factors should be respected to restore preoperative patellofemoral alignment and thus reduce the likelihood of anterior knee pain [14, 15]. In addition to coronal malalignment, however, there is another factor that has not yet been considered in current research. In parallel with the complexity of bony malalignment in patients with patellofemoral instability, increased femoral torsion (FT) might be another risk factor for anterior knee pain following TKA. It was shown that excessive FT negatively affects the outcome after surgical treatment of patients with patellofemoral instability [10]. Accordingly, osteotomies to correct axial and frontal plane malalignment have recently become popular [4, 7, 9, 11]. Furthermore, femoral rotational deformity was elaborated to affect patellar tracking and increase joint stress, resulting in accelerated femoropatellar joint degeneration [8, 26].

The purpose of this study was therefore to investigate whether excessive preoperative FT is associated with patellofemoral pain or instability following TKA. The hypothesis was that preoperative FT has no relevant influence on clinical outcomes of TKA patients. A second hypothesis was that as long as the frontal leg axis is aligned to roughly neutral, postoperative coronal alignment will not affect outcome either.

## Materials and methods

### Study population

All patients who received a primary total knee replacement (Medacta MyKnee GMK, Medacta International, Castel San Pietro, CH) at our institution as of 2019 were potential candidates for the study. Exclusion criteria were trauma (including patella fractures or dislocations), previous tumors, previous surgical knee procedures, chronic patellar dislocation, or malformation (or malunion after a fracture) of the affected limb. Complete preoperative radiographic (long-leg standing view, lateral and anteroposterior radiograph, axial view of the patella) work-up needed to be available too. For preoperative CT-based planning of patient-specific instrumented knee replacement, a computed tomography (CT) of the affected lower extremity was acquired using a specially developed protocol to scan the regions of interest (i.e. proximal femur, knee centre with distal femur and proximal tibia, ankle joint centre with distal tibia, distal fibula and talus).

Fifty-one patients of 210 showed increased FT of > 20° and were matched for age and sex to 51 controls (FT < 20°). Five study group patients (FT > 20°) refused to participate and 8 patients were lost to follow-up. Therefore, at final follow-up, two years after total knee replacement, the study population consisted of 38 matched pairs.

### Surgical technique

In all cases, patient-specific cutting blocks were used. Femoral and tibial cuts were performed to place implants perpendicular to the mechanical axis of the bone. However, deviations of bone cuts (and femoral component rotation) were made using PSI to ensure optimal gap balancing in some cases. No ligament balancing was performed in any case. Moreover, no effort was made to compensate for increased FT. The surgeon was not aware of the patient’s preoperative FT (FT was blinded). The femoral component was externally rotated with the sole goal of creating a rectangular and symmetric flexion gap. On average, external rotation was 2.5° (range 0–5°) from the posterior condylar axis. All patellae were denervated but no patella was resurfaced. All components were cemented.

### Clinical assessment

All 76 patients completed the final follow-up after two years. Kujala score and the patellofemoral scoring system for TKA were acquired preoperatively and two years after surgery [1, 24]. Patients reported outcome measurements independent of any medical staff. Postoperative clinical examination for objective scores was performed by one of the authors, and consisted of the following parameters: presence of patellofemoral crepitus (none, mild, moderate, severe), patellar tenderness (absent or present), presence of a J-sign, and quadriceps strength (M0-M5).

### Radiographic assessment

Parameters of interest were: FT, frontal mechanical leg axis, patellar thickness, patellar lateralization, patellar tilt, and tibial tuberosity-trochlear groove (TT-TG) distance.

Radiographs and CT of all included patients were analyzed on a picture archiving and communicating system (PACS) workstation by two independent observers (two orthopedic consultants). Parameters measured as Interclass Correlation Coefficients (ICCs) were calculated to determine the interobserver and intraobserver reproducibility, and are given in the methods section.

CT slice thickness was 2 mm for the proximal femur, 1 mm for the knee and 2 mm for the ankle joint, with increments of 1 mm, 0.5 mm, and 1 mm, respectively. All images were acquired using a clinical 40-slice or 64-slice CT scanner with image reformations using a bone kernel. CT examinations were performed with patients in supine position, with symmetric pelvis and straightened parallel legs.

Measurements (accuracy per pixel: 0.1 mm and 0.1°) were performed in a standardized technique as described in detail below. Regarding clinical relevance, outcome variables are given by one decimal.

#### Femoral torsion

CT-based measurement of femoral torsion was performed according to the method described by Murphy et al. [31]. Sutter et al. compared mean values and standard deviation of asymptomatic volunteers to patients with femoroacetabular impingement [39]. FT in the control group was 13° ± 10. Based on available data, the cut-off for increased femoral antetorsion was set to 20°. Inter and intrareader ICC for femoral torsion were 0.95 and 0.96 [5, 22, 42, 44].

#### Frontal mechanical leg axis

On long-leg standing radiographs taken preoperatively and one year after surgery, frontal leg axis was measured as the angle between the mechanical femoral and mechanical tibial axis, according to Strecker et al. [38]. Positive values indicate varus alignment and negative values indicate valgus. Inter and intrareader ICC for the measurement of frontal leg axis were 0.94 and 0.95.

#### Thickness, lateral displacement and tilt of the patella

The patellar thickness was measured from the anterior surface of the patella to the median ridge at the proximo-distal center of the median ridge, according to Iranpour et al. [21]. Lateral patella displacement was measured according to Zhang et al. (negative values indicating medial displacement) [45]. The patellar tilt was defined as the angle between a line across the posterior condyles and a line at the maximal patellar width, as previously described in various studies [23]. All parameters were measured on preoperative CT. Interreader ICC was 0.94, 0.84 and 0.83 for thickness, lateral displacement and tilt, respectively. Intrareader ICC was 0.95, 0.87 and 0.90, respectively.

#### Tibial tuberosity—trochlear groove distance

TT-TG measurement was performed on CT scans using the same methodology as described by Dejour [3]. Inter and intrareader ICC for the measurement of TT-TG were both 0.84 and 0.91, respectively.

### Statistical analysis

An a priori power analysis (*α* = 0.05, power level *β* = 0.90) revealed a minimum sample size of *n* = 40 (20 per group) to detect a minimum decrease of 5.41 points (minimal clinically important difference) in total HSS score with an increased FT, assuming a mean total HSS score of 95.1 points for satisfied patients after TKA according to Fan et al. [6]. The power analysis was conducted using G*Power (version 3.1; Franz Faull, Universität Kiel).

Descriptive statistics used frequencies and percentages to present the data. All parameters were tested using the Kolmogorov–Smirnov test for normality. When the criteria for normality were met, a 2-tailed paired *t* test was used. Otherwise, the Wilcoxon signed-rank test was applied. Chi-square test was used for non-parametric data. Spearman/Pearson correlation was used to identify a potential relationship between increased FT and clinical scores. A logistic regression analysis was used for odds ratio between clinical parameters, FT and postoperative coronal leg alignment. The level of significance level was set at a < 0.05. All the statistical analyses were performed using SPSS, version 22 software (SPSS Inc., Chicago, IL).

### Ethical aspects.

This prospective age and sex matched case–control study was approved by the Institutional Review Board and the local ethical committee (Zurich Cantonal Ethics Commission, KEK 2019–01749). It was conducted entirely at the authors’ institution. Informed consent was obtained of all patients.

## Results

No patient had preoperative patellar instability, and there was no pre or postoperative J-sign. There was no statistically significant difference between clinical scores two years after surgery between patients with normal and excessive FT (Table 1). Kujala score was 64.3 ± 16.7 versus 64.8 ± 14.4 (n.s.), and the patellofemoral score for TKA was 74.3 ± 21 versus 78.5 ± 20.7 (n.s.) for increased FT group and control group, respectively.

**Table 1:** Descriptive statistics of the demographical data and main parameters

	Increased femoral torsion group	Control group	*p* value
Demographical parameters			
Number of patients	38	38	
Gender			1
Female	30 (79%)	30 (79%)	
Male	8 (21%)	8 (21%)	
Age (years)	66.53 ± 8.4	66.82 ± 7.8	0.9
BMI (kg/m2)	29.4 ± 5.6	31.7 ± 6.4	0.1
Clinical parameters			
J-sign	–	–	1
Kujala score	64.3 ± 16.7	64.8 ± 14.4	0.9
Patellofemoral score for TKA	74.3 ± 21	78.5 ± 20.7	0.34
Anterior knee pain	2.2 ± 2.8	1.7 ± 2.6	0.463
Crepitus			0.082
None	16	21	
Slight	16	17	
Moderate	5	0	
Severe	1	0	
Radiographical parameters			
Femoral torsion (°)			
Preoperative	26.6 ± 6.1 (20.1–40)	10 ± 6.3 (− 6.8–19)	** < 0.001**
Postoperative	24.4 ± 6.1	7.3 ± 5.9	** < 0.001**
Frontal mechanical leg axis (°)			
Preoperative	1.1 ± 12 (− 18–25)	4.7 ± 8.7 (− 10–26)	0.185
Postoperative	− 1.61 ± 3.3	0.3 ± 3.4	**0.014**
Total correction (preop—postop)	2.7 ± 10.4	4.3 ± 7.4	0.445
Patellar thickness	20 ± 2.3	20 ± 1.8	0.875
Lateral displacement of patella	1 ± 3.2	0.5 ± 3.4	0.541
Patellar tilt	14.2 ± 6.8	12 ± 5.5	0.12
TT-TG distance (mm)	15.2 ± 4.1	11.9 ± 4.2	** < 0.001**

Continuous variables are shown as mean ± standard deviation (range), categorical variables are shown as number of patients and percentages of the total patient cohort. Significant values are **bold**

There was no correlation between preoperative FT and clinical scores (Table 2) two years after surgery. In the control group, FT was corrected downward more intraoperatively by external rotation of the femoral component (mean 2.8 ± 0.9° versus 2.2 ± 1.7° external rotation), not resulting in any change regarding correlation to clinical scores (Table 3).

**Table 2:** Correlation analysis of clinical data and preoperative radiographic parameters

Variable	Femoral torsion (preoperative)	Frontal leg axis (preoperative)	TT-TG (preoperative)
Kujala score	*r* = 0.128	*r* = 0.205	*r* = 0.085
	*p* = 0.272	*p* = 0.075	*p* = 0.466
Patellofemoral	*r* = 0.033	*r* = 0.159	*r* = − 0.023
score for TKA	*p* = 0.780	*p* = 0.170	*p* = 0.844

**Table 3:** Correlation analysis of clinical data and postoperative radiographic parameters

Variable	Femoral torsion (postoperative)	Frontal leg axis (postoperative)	Total frontal leg axis correction
Kujala score	*r* = 0.128	*r* = 0.087	*r* = 0.196
	*p* = 0.271	*p* = 0.456	*p* = 0.092
Patellofemoral	*r* = 0.006	*r* = 0.157	*r* = 0.073
score for TKA	*p* = 0.961	*p* = 0.180	*p* = 0.532

All other radiographic parameters were similar between both groups (Table 1), except TT-TG distance which is due to its correlation to FT. Subjects from the control group showed 3.6° more varus preoperatively (n.s.). After surgery, frontal leg axis was more neutrally aligned (difference 1.6°). No correlations of clinical outcomes and preoperative/postoperative frontal leg axis or total leg axis correction were found (all n.s.) (Table 2 and 3). Logistic regression analysis found no lower clinical scores in patients with a postoperative valgus (n.s.) (data not shown).

## Discussion

The most important finding of this study is that excessive preoperative FT is not associated with patellofemoral pain or instability following TKA.

Previous reports advocated for restoration of individual patellofemoral tracking with the aim of decreasing the risk of anterior knee pain [14, 15]. As the role of excessive FT in TKA has not yet been explored in literature, the main goal of this prospective study was to answer the question whether excessive FT should be corrected in TKA, as it is increasingly performed in patients with patellofemoral instability [4, 7, 9, 11, 18, 19]. However, no correlation between FT and scores which are specific for the patellofemoral complaints could be found. Moreover, results were independent of the extent to which FT was compensated by means of external rotation of the femur component to balance the gaps.

Other morphologic factors influence patellofemoral tracking such as coronal leg alignment and TT-TG [20]. To date, the most current philosophy is to align the prosthesis guided by individual constitutional phenotype to restore normal knee kinematics and function. Kinematic alignment, as proposed by Howell in 2006, places no restrictions on the patient’s anatomy and post-operative correction [13, 16, 17, 25]. There are, however, reports that postoperative valgus alignment should be avoided [36]. Only in patients with preoperative non-varus alignment, Slevin et al. found a significant correlation between neutral limb alignment and higher clinical scores [36]. Nevertheless, no influence of overall leg alignment on postoperative outcome could be shown in other studies [35]. This is consistent with the here presented data, as no correlation to patellofemoral scores was found despite a significant amount of postoperative valgus knees. Nonetheless, it was demonstrated that femoral valgus alignment negatively affects patellofemoral tracking in that the patella showed increased bone tracer uptake with femoral valgus [37]. Arguably, this effect might be only subclinical if valgus does not exceed 5°—or symptoms are prevented by intraoperative patellar denervation with electrocautery [12, 41].

This study has several limitations. First, preoperative patellofemoral grade of osteoarthritis was not assessed and compared between groups. It would have been interesting to examine the condition of the patellofemoral cartilage intraoperatively to determine the extent to which this affects anterior knee pain. A preoperative MRI was available for 15 patients in the increased FT group and 12 controls only. However, although the results were certainly underpowered, there was no difference between the lateral or medial retropatellar cartilage condition according to the ICRS classification (n.s.). This is in accordance to a recent published paper that found no association between clinical outcomes and visual grading of patellar cartilage degeneration after TKA without patella resurfacing [32]. Furthermore, no postoperative CT scan was available to assess postoperative TT-TG distance, patellar tilt or displacement, which could have confounded the scores. TT-TG distance, for example, is expected to significantly decrease independently of preoperative mechanical leg axis [46]. Further study is however warranted to evaluate alteration of TT-TG distance as a potential cause for postoperative patellofemoral maltracking and pain. This would have gone beyond the scope of this study. Furthermore, it must be added that knowledge and surgical technique of TKA have improved substantially over the past years. Accordingly, the rate of outliers in terms of component placement and postoperative frontal axis deformity has become low, which makes thorough research on this topic somewhat difficult [33]. Finally, standard deviations of clinical scores were higher in this study cohort than those used for the a priori power analysis, implying that this study may still be underpowered. However, given the nearly identical results, no difference is expected in a larger patient cohort.

## Conclusion

If the leg axis deformity is corrected to a roughly (± 5°) neutral alignment during cemented TKA, including patellar denervation, then excessive FT was not associated with patellofemoral pain or instability.
